# Examining Unmet Need for Adults Presenting Self-Harm and Suicidality in Jaywick

**DOI:** 10.1192/bjo.2026.11075

**Published:** 2026-06-30

**Authors:** Emma Kaminskiy

**Affiliations:** Anglia Ruskin University, Cambridge, United Kingdom

## Abstract

**Aims:**

Adults living in deprived coastal communities face disproportionately high rates of self-harm and suicide, yet little is known about the barriers they experience when attempting to access preventative and mental health support. This study explored how Jaywick residents navigate existing support pathways, identified key barriers and unmet needs associated with accessing services for self-harm and suicidality, and examined approaches that may strengthen engagement and early intervention within this coastal setting.

**Methods:**

Two stakeholder workshops were conducted with 14 participants from statutory health providers and community and voluntary organisations operating in Jaywick. Workshops were audio-recorded, transcribed verbatim, and analysed using reflexive thematicanalysis. Discussions focused on experiences of service navigation, barriers to accessing care for self-harm and suicidality, and opportunities for system improvement.

**Results:**

Three overarching themes were identified. First, access barriers: digital exclusion, low literacy, and poor signposting impeded residents’ ability to access mental health support, leaving many dependent on others for referrals. Second, social and cultural dynamics: deep-rooted mistrust of statutory services, normalisation of symptoms, and strong community tribalism hindered help-seeking and engagement. Third, vulnerability and risk: entrenched cycles of poverty, addiction, trauma, and isolation were prevalent, with migrants and refugees experiencing added disadvantages. Fragmented service provision meant that those with co-occurring needs frequently fell between service thresholds and were often only supported at crisis point.

**Conclusion:**

Barriers to accessing support for self-harm and suicidality in Jaywick are complex, intersecting, and reinforced by long-standing structural inequities. Enhancing trust, embedding services locally, and reducing digital exclusion are essential to improving early intervention and access. Co-produced, community-based models, including health navigation and outreach approaches, offer promising routes to addressing the significant unmet needs within this coastal community.

